# The C++ programming language in cheminformatics and computational chemistry

**DOI:** 10.1186/s13321-020-0415-y

**Published:** 2020-02-07

**Authors:** Dmitrii Rassokhin

**Affiliations:** Janssen Research & Development, LLC, 1400 McKean Road, Spring House, PA 19477 USA

**Keywords:** Programming languages, C, C++, Scientific computing, Computational chemistry, Cheminformatics

## Abstract

This paper describes salient features of the C++ programming language and its programming ecosystem, with emphasis on how the language affects scientific software development. Brief history of C++ and its predecessor the C language is provided. Most important aspects of the language that define models of programming are described in greater detail and illustrated with code examples. Special attention is paid to the interoperability between C++ and other high-level languages commonly used in cheminformatics, machine learning, data processing and statistical computing.

## Introduction

In recent years, a plethora of high-level domain-specific and general-purpose programming languages have been developed to greatly increase the productivity of programmers working on various types of software projects. Scientific programming, which used to be dominated by Fortran up until about mid-1980s, now enjoys a healthy choice of tools, languages and libraries that excel in helping solve all types of problems computational scientists and scientific software developers deal with in their everyday work. For example, MATLAB is widely used for numerical computing, R dominates statistical computing and data visualization, and Python is a great choice for a wide range of scientific applications from machine learning and natural language processing to typical cheminformatics tasks like chemical structure search and retrieval, virtual compound screening and molecular property prediction, just to name a few. However, among modern high-level programming languages, C++ plays a special role of being the language that de-facto dominates modern scientific software development, even though, at first glance, this may not be so obvious. In this paper, we will briefly describe the history of C++ and focus on its main characteristics that make it so special.

## Brief history of C and C++

The predecessor of C++, C was developed in the early 1970s by Dennis M. Ritchie, then an employee of Bell Labs (AT&T), when Ritchie and his colleagues were working on Unix, a multi-user time-sharing operating system for mainframe computers. Early versions of this now ubiquitous operating system were written in architecture-specific non-portable assembly languages. As Unix was being further extended and gained popularity, the developers realized the need to re-write parts of it in a platform-independent high-level programming language to make the codebase more manageable and easily portable to different computer architectures. Back then, Fortran was one of the most commonly used high-level languages. Being the language of choice for numerical computing, Fortran circa early 1979s was not suitable for low-level programming due to its verbose flow control structures and the absence of direct memory access operations. Fortran was also ill-suited for non-numerical computing, which typically involves defining complex data structures and operations on them, while languages designed for symbolic computing and list processing, such as Lisp, the second-oldest high-level computer language after Fortran, were quite difficult to master, and often required specialized and very expensive hardware to achieve acceptable performance [1]. It is remarkable that one of the first very large and complex cheminformatics software packages, an interactive computer program designed to assist planning syntheses of complex organic molecules called LHASA (Logic and Heuristics Applied to Synthetic Analysis), was largely written in Fortran and contained nearly 30,000 lines of very complex Fortran code [2, 3].

A better alternative for further Unix development was the programming language B, which was derived from BCPL in the 1960s by Ken Thompson for coding machine-independent applications, such as operating systems and compilers for other languages. The B language can be considered the direct predecessor of C. B was much more suitable for the operating system development compared to Fortran, since it provided constructs that map efficiently to typical machine, had a clear and concise syntax and supported efficient direct memory access operations. The main shortcoming of B was the lack of support for data types. In fact, it supported only one type, the architecture-dependent computer word treated as an integer. Therefore, in B, operations on data types other than the machine word (such as, for example, single-byte characters or structures composed of fields) were difficult to implement in a portable way. There shortcomings also made B totally unsuitable as a general-purpose programming language. In the early 70s, Dennis M. Ritchie gradually added support for primitive (integer and floating-point numbers, and characters) and complex (user-defined structures) data types to B and cleaned up its syntax. Eventually, the improved B differentiated from the original B so much that it become a different language, which was half-jokingly called C after the next letter of the English alphabet. In 1978, the first edition of the famous “The C Programming Language” book written by Brian Kernighan and Dennis Ritchie was published [4]. The version of the C language described in the book is often referred to as K&R C, after the book authors. The C language quickly gained popularity among operating system and device driver developers. Subsequently, most of the Unix components were rewritten in C. Due to the relative simplicity, portability, and efficiency, the popularity of C soon went far beyond its original intended purpose of operating system development, and it became one of the most commonly used general-purpose programming languages for a range of applications from device drivers, microcontrollers and operating systems to videogames and high-performance data analysis packages.

In 1983, a committee formed by the American National Standards Institute (ANSI) to develop a standard version of the C language based on the K&R C. ANSI published the standard definition in 1989 and is commonly called “ANSI C”. Subsequently, the ANSI X3.159-1989 C standard has undergone several revisions, the most recent of which (informally named C18) is ISO/IEC 9899:2018 [5].

In the 1970, the object-oriented programming (OOP) paradigm was quickly gaining popularity. Simula 67, the first programming language to support the OOP, was developed primarily for discrete event simulation, process modeling, large scale integrated circuit simulations, the analysis of telecommunication protocols and other niche applications. In 1979, Bjarne Stroustrup, while working towards his Ph.D. in Computer Science at the University of Cambridge, England, used Simula 67 to implement calculations for his research and found the OOP paradigm to be very productive, but all its existing implementations inefficient. At that time, C had already become one of the most used general-purpose programming languages, so Stroustrup got a brilliant idea of adding OOP features to C and started his work on “C with Classes”, the superset of K&R C, which would support object-oriented programming while preserving the portability, low-level functionality and efficiency of C [6]. Early implementations of C with Classes were translators that converted “C with Classes” code into the standard K&R C, which could be compiled by any available C compiler. “C with Classes” was extended by adding, among other important features, improved type checking, operator overloading, and virtual functions. In 1983 Stroustrup renamed “C with Classes” to C++. The ++ operator in the C language is an operator for incrementing a variable, which reflected Stroustrup’s notion of C++ being the next generation of the C language. In 1986, Stroustrup published his famous book called *The C++ Programming Language* [7], which became the de-facto language reference manual. Very soon, C++ started gaining a widespread popularity in the developer community, and several good quality C++ compilers and libraries become available for practically all major computer platforms and operating systems.

Probably, the most important C++ release was C++ 2.0 in 1989, documented in *The Annotated C++ Reference Manual* by Ellis and Stroustrup [8]. C++ 2.0 was a full-fledged object-oriented language with support for multiple inheritance, abstract classes, static member functions, constant member functions and protected class members, templates for generic programming, exceptions for structured error handling, namespaces, and a Boolean type.

The next important release came in 2011, when the C++11 standard was published. C++11 has been augmented with several features affecting runtime performance, most importantly, the “move constructor”, which eliminated the bane of earlier C++, the costly and unneeded copying of large objects when they are passed to or returned from functions by value. C++11 also included a number of significant features for producing terser, more readable code; chief among these are auto variables (removing the need for detailed variable declarations while preserving type safety) and range-based “for” loops (allowing looping over the elements of a container with an almost Python-like syntax).

After the long delay to reach C++11, the C++ Standard Committee has been updating the C++ standard every three years. In 2014, the C++14 standard was published, followed by C ++17 in 2017, which, at the time of writing this article, is the most recent revision of the ISO/IEC 14882:2017 standard for the C++ programming language [9]. The next standard release is planned for 2020. The language is quickly evolving to improve the code readability and expressive power. For example, lambda-expressions with closures introduced in C++11 and enhanced in C++14 [10], obviously inspired by functional programming languages like Haskel and F#, make it possible to pass function-like objects to generic methods, such as sorting, searching and filtering, which considerably shortens the code using these methods without sacrificing the performance. Latest versions of C++ make it easier to write portable code that takes advantage of modern multicore computer architecture by providing facilities to create and manage sequences of instructions executed concurrently with other such sequences (commonly referred to as “threads”) and synchronize memory accesses among different threads running in parallel.

As of 2019, C and C++ remain extremely popular programming languages for a wide range of applications [11]. In scientific programming, including cheminformatics and computation chemistry, scripting languages like Python (general-purpose) or R (statistical applications, predictive modeling and machine learning) have seen the explosion of popularity in recent years; however, as it will be discussed further below, it is a very common scenario when Python or R are used to assemble computational workflows from components of numerical, machine learning, statistical, cheminformatics, molecular mechanics and other specialized packages written in C or C++.

C++ programmers enjoy a great ecosystem of development tools. Compilers, debuggers, and integrated development environments, both free and commercial, are easily available for all modern computer platforms. The competition between the two major open source C++ compilers GCC [12] and Clang [13] has led to rapid progress in the quality of the object code produced and, importantly, the utility of the feedback provided to programmers in the case of errors, warnings, and diagnostic messages. Both GCC and Clang are widely and easily available on Linux and MacOS computers. Microsoft Windows does not, by default, come with a C++ compiler, but one can readily download the Microsoft Visual Studio integrated development environment, which includes a C++ compiler, runtime libraries and tools, directly from Microsoft [14]. Compilers that generate very efficient code targeting specific hardware are also available from various vendors. For example, Intel C and C++ compilers are highly optimized to processors that support Intel architectures [15]. Sophisticated integrated development environments that offer built-in C/C++ code editors with syntax highlighting, context-sensitive help, powerful debugging, profiling and refactoring tools, visual interface designers, and various features that facilitate large developer teams working together on large-scale software projects are readily available, both commercial (such as Microsoft Visual Studio [14] and Visual Studio Code [16] from Microsoft or CLion [17] from JetBrains and open-source, such as, for example, a widely-used Eclipse CDT [18]. Libraries of C and C++ code are available for every programming task imaginable, from low-level hardware control to machine learning and natural language processing.

## C++: Basic language features

Let us first discuss basic features of C++, which it inherited from C and which are not related to advanced concepts like object-oriented or generic programming. It should be noted that modern C is not a true subset of modern C++, and a modern C++ compiler will not compile most non-trivial programs written in modern C without at least some minor modifications. However, for the purposes of this paper we can consider modern C++ to be an extension of “classical C with better type safety and without some relatively rarely used features”. In this section, for brevity, C++ will mean “C or C++”.

### C++ is primarily a compiled language

Before it can be executed, an entire program in C++ must be “built”, that is, translated to the target machine’s native instructions by a program called *compiler* and linked with external pre-compiled libraries by a program called *linker*. High-quality compilers perform extensive local and global code optimization and produce very efficient and compact code. Compiled programs do not need any additional runtime environments to be present on target computers in order to be executed. Compare this to interpreted languages, such as Python, or languages that are typically compiled into and delivered to users as platform-independent intermediate code, just as Java. Python code needs a Python interpreter in order to be run, and programs compiled into the intermediate Java byte code need a Java runtime environment to translate the intermediate code into the host machine instructions at runtime. A large C++ program can take significant time to compile, since every single line of its source code has to be processed by the compiler, regardless of whether it will actually be executed during an invocation of the program. This slows down the development cycle, but typically results in more reliable code, as the compiler can catch many errors at compile time, thus avoiding unpleasant “runtime error” surprises so typical for interpreted languages like Python. Yet another downside of a compiled language is that the executable files produced by a compiler from source code are not portable and will only run on the target platform (that is, the hardware plus the operating system) for which they are compiled, or a binary-compatible platform. Special care must be taken when writing C++ code, specifying compiler options and choosing code libraries to link with to satisfy specific binary compatibility requirements (see, for example, the Wikipedia article on binary compatibility [19] and Red Hat Enterprise Linux 7: Application Compatibility Guide [20] just to get an idea of how complicated the issue of binary compatibility can be). In order to port a C++ program or library to a different platform, the source code must be re-compiled specifically for that platform. Since nowadays C++ compilers exist for all major computer platforms and operating systems, generally, C++ source code is highly portable. However, complex programs written in C++ using non-standard or poorly supported language features or having dependencies on code libraries that have not been widely ported, or relying on specific machine or OS features, such as, for example, the machine word size, byte order, or the support for certain specific CPU instructions, can be extremely difficult to port and may require making changes at the code level by an experienced C++ programmer. There exists a very useful online tool called Compiler Explorer [21], which can compile snippets of programs in many programming languages including C++ with various compilers and options interactively and visualize the machine code output. This makes it a great teaching tool, which can also be used low-level code optimization.

It should be noted that the C++ language standard does not prescribe that a program in C++ must first be compiled in its entirety into an executable file containing the target platform machine instructions before it can be run. C++ interpreters that allow the execution of C++ code in the interpreted and/or interactive mode line-by-line do exist (for example, Cling [22]), but the very nature of the language, in particular, the static typing, does not play along well with the interactive read-evaluate-print-loop (REFL) execution mode, so C++ interpreters remain very specialized niche tools for quick prototyping and compiler development.

### C++ is imperative

Imperative programming is a programming paradigm in which a program consists of statements (or commands to the computer) that change a program’s state. Imperative programming focuses on describing how a program operates, and imperative code closely maps to machine code native to the computer. At the low level, the program state is defined by the contents of memory, and the instructions in the native machine language of the computer prescribe the hardware how to change the data in memory. Higher-level imperative languages abstract away platform-specific instructions, for example, use variables instead of memory locations and statements written in human-readable notation rather than instruction codes, but still follow the same pattern.

Compare the imperative to the declarative paradigm, which focuses on what the desired result should be and leaves it up to the execution engine to “decide” how to obtain it. A common declarative language familiar to most data scientists is SQL (Structured Query Language), which is designed to manage data stored in a relational database system, such as Oracle or PostgreSQL. For example, a typical SQL ‘select A, B, C from Table1 join Table2 on Table1.K=Table2.FK’ data query statement describes **what** records to retrieve from which tables in a relational database, but does not instruct the database engine how to do this, and the implementations of SQL query processors can be vastly different between different database engines.

### C++ is procedural

A procedural programming language is an imperative programming language which supports the concept of procedures and subroutines isolating segments of code into reusable units that can be “called” to perform individual tasks. Procedures and subroutines are known as *functions* in C or C++. A C++ function can take zero or more parameters (sometimes called arguments) and return zero or one value.

### C++ supports structured programming

Structured programming languages provide intuitive mechanisms to control the flow of a program (that is, the order in which statements are executed). The structured flow control statements in C++ are similar to the ones found in many other structured programming languages. These are *if/else* for implementing branching logic, and *for*, *while*, and *do/while* for implementing iterations (loops). C++ does have the notorious *goto* statement that can be used to pass control to an arbitrary location within a function in a “non-structured” way, but it is rarely used.

### C++ has lexical variable scope

As most modern languages, C++ uses lexical scoping for variables and functions. A variable or function in C++ may only be referenced from within the block of code in which it is declared. The scope is determined when the code is compiled. The opposite of lexical scope, dynamic scope refers to scope of a variable defined at run time and depending upon the program state when the name of a variable is encountered.

### C++ is statically typed, but not type-safe

The compiler does the type checking when a C++ program is being compiled. This helps detect common programming mistakes. In dynamically typed languages (such as, for example, Python or JavaScript) the types of variables and functions are checked at run-time, which allows for extra flexibility and sometimes shortens the code, but often results in runtime errors when an operation or function is applied to an object of inappropriate type. It should be noted that C++ is not a type-safe language. C++ compilers will allow many operations on typed variables that might lead to undefined behavior or errors, but usually the programmer must “let the compiler know” his or her intension, for example, by “casting” a pointer to a memory location to a certain type. This comes very handy in low-level programming where efficient access to hardware is a must, but the programmers are expected to know what they are doing, since errors arising from unsafe type conversions are notoriously difficult to debug and are often platform-dependent.

### C++ has facilities for low-level memory manipulation

C++ provides operations on pointers to arbitrary memory locations, which makes C++ a perfect choice for programming operating systems, embedded systems and device drivers. For instance, a peripheral input/output device driver may map (or associate) the memory and registers of the controlled device with certain reserved addresses [12]. To control the device, the device driver assigns values having special meaning according to the device specifications to those reserved memory locations. For example, the following statement in the driver’s code (assuming it is implemented in C or C++) sets the byte at the memory location 40008000 (in hexadecimal notation) to 1.
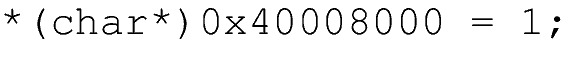


The **char** data type in C/C++ is the smallest addressable unit of the machine (one byte consisting of eight bits on most modern computers). The (char*) is the **type cast** operator telling the complier to interpret 0x40008000 as a pointer to a byte at the memory location 0x40008000, and the prefix * (the asterisk character) is the **pointer dereferencing** operator used to access (read or write) the value stored at that location.

Manipulating data via memory pointers in C++ is a very common practice not only in low-level system programming, but also in the implementation of a wide variety of algorithms and data structures with minimum possible overhead. Common vector-type data structures such as vectors, matrices and character strings are efficiently represented in C++ by contiguous memory blocks containing data of a certain type, and C++ provides very terse syntax for operations on these memory blocks. For example, finding the position of a character in a zero-terminated C string using C pointer operations can be done with just one line of code, the *while* loop in the code snippet shown below:
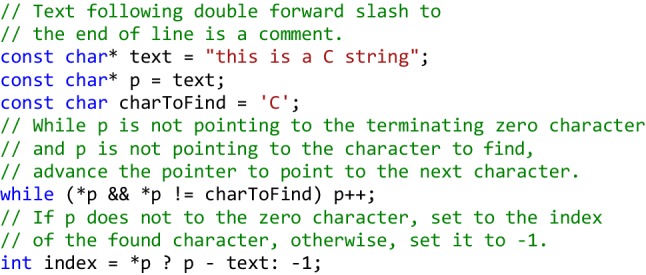


### C++ has deterministic memory allocation and de-allocation

The lifetime of objects in C++ is deterministic and defined by the programmer. This eliminates the overhead of “garbage collection”, where the runtime environment (such as, for example, the Java Virtual Machine or Python interpreter) must trace the lifetime of objects during the program execution and, when an object is no longer used, free up the resources associated with it [23]. It also allows placing an object at a specified memory address. This makes C and C++ particularly suitable for writing code for resource-limited systems, such as real-time systems and microcontrollers. Below is an example illustrating C/C++ deterministic heap and stack [24] memory management:
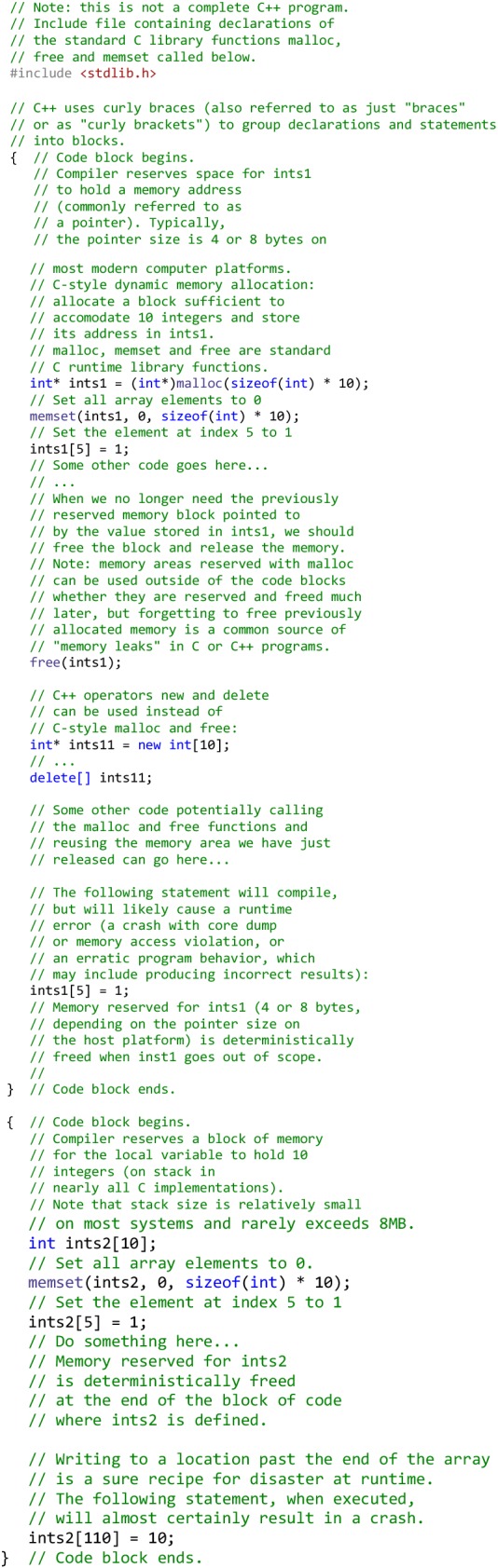


The ultimate efficiency of C++ achieved via direct access to memory via pointers, explicit deterministic memory management and a very close mapping of C++ language constructs to hardware makes C++ the language of choice in high-performance scientific computing, when implementing CPU- and memory-intensive algorithms, for example, molecular mechanics modeling, machine learning and statistical analysis of very large volumes of data. The efficiency comes at a cost though. Programmer’s errors, such as accessing an array out-of-bounds, or forgetting to properly initialize a pointer, result in random program crashes or unexpected behavior, which, in complex code, can be extremely difficult to locate and fix. Intentionally (for efficiency) or unintentionally skipping checks for common manual memory management errors, such as buffer overflow, in C or C++ code is a security vulnerability that has been often exploited by computer viruses, ransomware and other types of malware [25]. Numerous static and dynamic code analyzers and debuggers exist that help programmers detect memory management errors in C++ code, such as, for example, the GDB debugger [26] and the Valgrind toolkit [27]. Nevertheless, even with the help of the most sophisticated C++ compilers and development tools, memory management errors in non-trivial C and C++ code are hard to avoid even for experienced programmers. As it was mentioned above, many high-level languages, such as Python or Java, provide automatic memory management with ‘garbage collection’ and disallow or restrict direct memory access via pointers, thus eliminating the possibility manual memory management bugs altogether. However, automatic memory management has substantial performance implications and makes these languages unsuitable for low-level programming.

### C++ is a high-level language with low-level functionality

C++ offers the ample means for programmers to express their ideas at the high or low level of abstraction, depending on the specific task at hand. C++ (especially, its C subset) has very little runtime overhead and, as it was already mentioned above, uses deterministic explicit memory allocation/deallocation. If desired, a C++ program can be written in a minimalistic ‘portable assembly language’ style to effectively control the hardware, for example, when programming device drivers. At the same time, C++ allows coding in terms of abstract computer science concepts, such as functions, programmer-defined types and operators, generic types, lambda-expressions and closures, which makes it suitable for implementing complex algorithms with non-trivial execution flow logic, for example, graphical user interfaces and compilers. In scientific programming, C++ is often used as a high-level object-oriented language, taking full advantage of its expressive power. High-level features of C++ will be described in more detail below in the sections of this paper discussing object-oriented and generic programming.

### C++ has pre-processor, which adds some meta-programming capabilities to the language

Before being passed to the compiler, C++ code is pre-processed to expand the so-called pre-processor directives. The most common directives in C++ are expandable macros, file inclusion and conditional compilation directives. A detailed description of these is beyond the scope of this paper, but the interested reader will find a few examples of pre-processing directives in the *Hello, World* code below. They can be identified in the source by the # (hash) character that marks the beginning of a directive.

### Hello, World in C

Before we address more advanced concepts related to object-oriented and generic programming in C++, let consider a working example of a simple program that demonstrates the “C subset” of C++. Code below shows a slightly extended and commented version of the traditional “Hello, World!” program that can be run from a command line to display “Hello, World!” or “Hello, <*someone*>”, depending on the command-line arguments it is invoked with. Note the #include<*filename*> directive that includes the contents of the **header** file identified by the *filename* into the current source file.
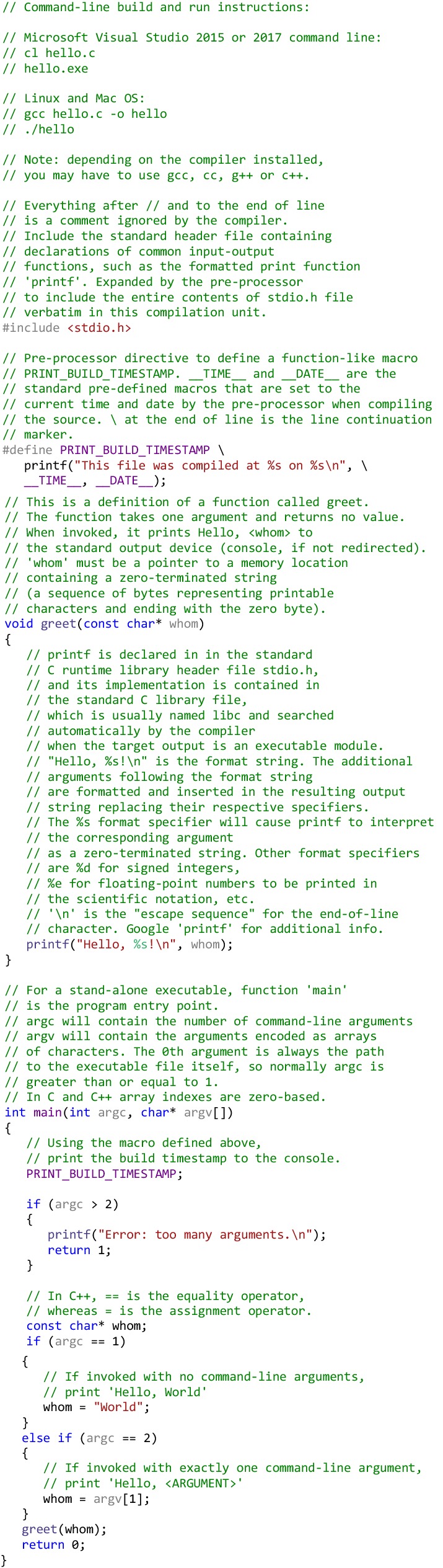


The program also illustrates the typical **compile/link/run** C++ program execution pattern. In order to produce an executable file from the above source code, one has to *compile* it to an intermediate module and *link* the module with standard and custom libraries that contain pre-built implementations of functions used in the source but not defined there. The above example is very simple and depends only on the standard C runtime library for the program initialization routines and the implementation of the *printf* function, so it can be compiled and linked to produce the executable on most modern Unix-like systems (Linux, Mac OS X, FreeBSD, AIX and others) with a very simple command:
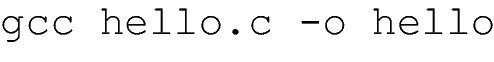


When run with the above command-line options, compiler will invoke the linker and link the standard C runtime libraries automatically to produce the executable module. All modern Unix-like systems come with a C/C++ compiler, but, depending on the specific OS version and distribution, you may have to substitute cc, C++ or g++ for gcc. If the compiler discovers one or more syntax errors in the source code during the compilation, it will report them to the programmer and generate no executable file. Running the resulting executable from the command line will output “Hello, World!” or “Hello, *argument*!”.
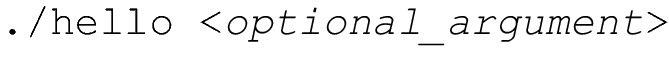


It should be pointed out again that the compiled executable file contains the machine code for the target platform and **does not need an interpreter or runtime environment in order to run**. However, it is platform-specific and will not run on hardware and operating system other than the one for which it was built, or its emulator. This contrasts with interpreted languages like Python, where the interpreter translates the program source code into the machine instructions at runtime and immediately executes these instructions. Programs in many languages including C or C++ may be either compiled or interpreted, so being “compiled” or “interpreted” is not an essential property of a language per se. The overwhelming majority of C and C++ implementations are compilers rather than interpreters though. The structure of C/C++, primarily, the static typing, makes its use as an interpreted language quite cumbersome and does not realize its full potential as a language for system programming and high-performance computing.

## C++: Object-oriented and generic programming

### Basic ideas

As mentioned in Short history of C and C++ section above, one of the distinctive features of C++ is its extensive support for objective-oriented and generic programming. A programming language that serves the purpose of representing a programmer’s ideas in an understandable form to the computer dictates not only a way of representation but also, to a considerable extent, the ideas themselves. All programming languages consist of certain systems of terms and concepts set in a framework into which the programmer subconsciously “squeezes” the program he or she creates as early as during the design stage. In other words, the way a program is coded dictates to a considerable extent the way the program is designed. One cannot set yourself free from a language’s dictates, but this is not necessary. The desired solution to this situation is to use a computer language that closely supports the system of concepts on which we base our vision of the world—thus, the path from design to implementation will be easier and the productivity of the labor involved will increase.

This is exactly what object-oriented programming (OOP) suggests. OOP demands an object-oriented approach to program design—the so-called object-oriented design (OOD)—that, in turn, successfully exploits our natural human abilities of classification and abstraction. For instance, in speaking the word “window” we imply something can be seen through it. Both a window in a house through which we view a street and a “window” on a computer screen, which is just a (usually rectangular) area with distinct boundaries containing various graphical elements drawn by a program possess that property. So, these window “instances” can be thought of as belonging to a class (or type, or concept) called “Window”. Classes and objects, inheritance, and hierarchy are intrinsic to human thinking and intuitively understood.

OOD and OOP are really the processes for the design and creation of a specific world—a program—inhabited by objects that are born, change their internal state, interact with each other, and die. And OOP requires the programmer become first a creator who considers a program not as a subsequence of actions but as a specific world living its own life.

Rather than thinking in terms of data and procedures the OOP paradigm encourages thinking in terms of interacting objects that possess certain *properties* and exhibit certain *behaviors*.

Let us consider a specific example from the field of cheminformatics. Practically all cheminformatics toolkits support chemical structure (or molecule) representation based on graph theory. The most natural representation of a molecule is a graph where the atoms are encoded as the graph nodes and the bonds are the graph edges. In the “traditional” non-OOP approach, one would design this program by first defining a data structure that represent the basic graph, for example, as a N×N square symmetric connection matrix M, where N is the number of atoms in the molecule. If atom *i* is connected to atom *j*, the corresponding elements of the matrix $M_{ij}$ and $M_{ji}$ will contain 1, otherwise, they will contain 0. In addition to the connection matrix, one will need to define data structures to represent properties of each atom and bond, for example, the atomic number and bond type. Having defined the data structures, the developer would define a set of procedures to operate on these structures, for example, to add an atom to the molecule, connect an atom to another atom with a bond, determine how many atoms and bonds are in a molecule, read from and save a molecule into a structure file, and so on. Data in such a program are, so to speak, low-men-on-the-totem-pole, being considered only as a sphere of action for functions.

The OOP paradigm encourages a completely different mode of thinking, based on the **data abstraction** and **encapsulation**. When designing code to represent molecules in the OOP style, one should focus on data fields representing a *state* of a molecule and common operations that can be applied to all *instances* of a molecule. In this train of thought, molecules are represented as *objects* (or instances) of the abstract data type (or “*class*”, using C++ terminology) Molecule. In this context, ‘abstract’ means that the type is defined in terms of operations that can be applied to it and the expected behavior of these operations rather than its internal structure and details of its implementation. Bundling (or **encapsulating**) the data and methods that operate on that data in one conceptual unit—a class,—exposing only operations that define its behavior to the “outside world” and hiding implementation details greatly facilitates code reusability and modularity. For example, in the code snippet below, the adjacency matrix-based molecular graph representation can be replaced with an alternative representation based, for example, on a graph edge list. After such a change, any dependent code using only public methods and fields of Molecule can be re-compiled and used with no modifications.
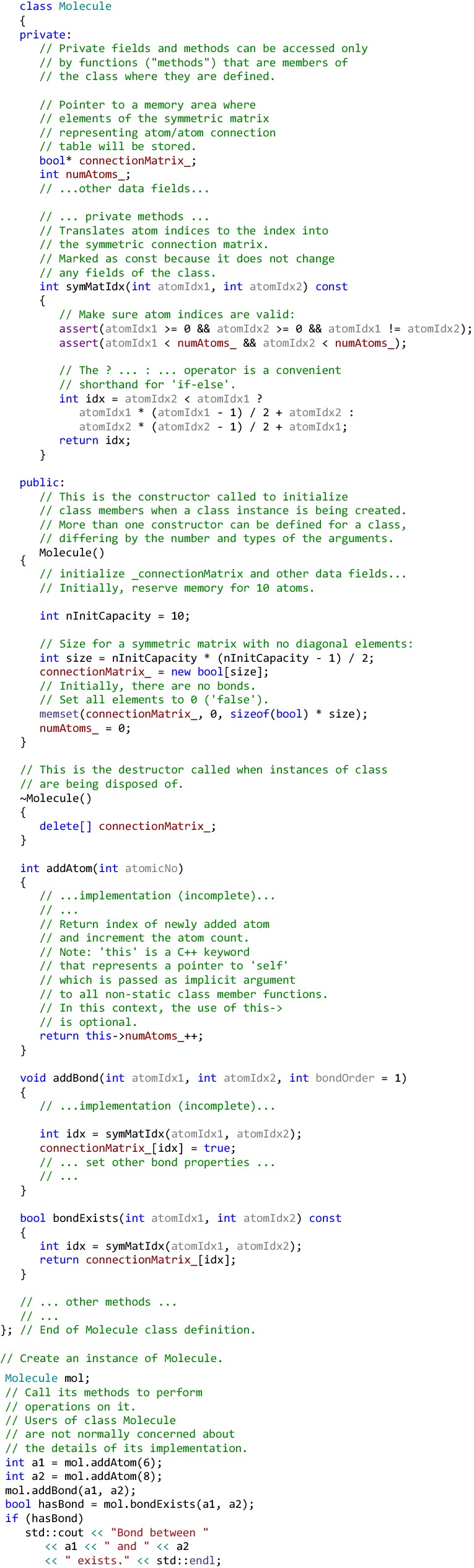


**Data abstraction** is one of the key concepts of OOP and OOD. Other key concepts on which OOP is based are **inheritance**, **composition** and **polymorphism**.

**Inheritance** means deriving more complex data types from simpler ones. C++ offers special mechanisms to successfully exploit that idea. A derived class “inherits” properties and behaviors of its ancestor classes, while adding new properties and behavior. Using class inheritance, one can design complex classes from the general to the specific. Using our Molecule class as an example, it would be natural to think of it as a superclass of a base class called Graph, inheriting the internal representation and graph algorithm functions and adding features specific to molecules, such methods to access and change properties of atoms and bonds in a molecule, compute basic molecular properties, etc.

**Composition** in OOP is yet another method of building complex types, alternative to inheritance. Types extended via composition *contain* instances (or pointers to instances) of other classes implementing additional functionality rather than *deriving* from those classes. For example, if we want molecules to be able to encode themselves into the SMILES linear notation [28], we can derive a class called, for example, SmilesEncodableMolecule from the base class Molecule and implement the method that will be returning SMILES-encoded molecules called, for example, getSmiles(), plus all additional methods and fields needed for its implementation in the derived class. Alternatively, using the composition-based approach, we can re-design the base class Molecule to have a container-type data field to hold pointers to various encoders, develop a class that represents a SMILES encoder, and add an instance of the SMILES encoder to an instance of Molecule at runtime. A detailed discussion of composition vs. inheritance is beyond the scope of this paper, and an interested reader can refer to the Wikipedia article [29], which has multiple references to publications where the pros and cons of either approach in various development scenarios and programming languages are debated.

**Polymorphism** is a Greek word meaning “having many shapes”. Applied to OOP, this term is usually regarded as the property of an object to respond to an operation according to the object’s type, even if its type is unknown at compile time. For example, we can define types Square and Circle as deriving from the base type Shape and pass a reference or a pointer to an instance of type Shape to some function as an argument (for example, that function may be defined as void f(Shape* s)). Inside that function, we would call the function area() declared in the base type Shape and defined in types Square and Circle. Even though at the compile time the compiler would have no information on the exact type of the object that can potentially be passed to the function f (as long as it derives from the base type Shape), it will generate the code to invoke the correct type-specific implementation of the function area(), defined either in type Square in type Circle, depending on the actual type of the object, and applying the correct formula to calculate the area of the object.

**Generic programming** is a style of programming in which algorithms are written in terms of to-be-specified-later types that are then instantiated when needed for specific types provided as parameters [30]. C++ provides very effective template-based mechanisms for generic programming, which make the generalization possible without sacrificing efficiency, since the compiler generates the type-dependent code, so the type determination and the type-dependent function binding do not have to happen at the runtime. A trivial example of defining and instantiating a *function template* is shown below.
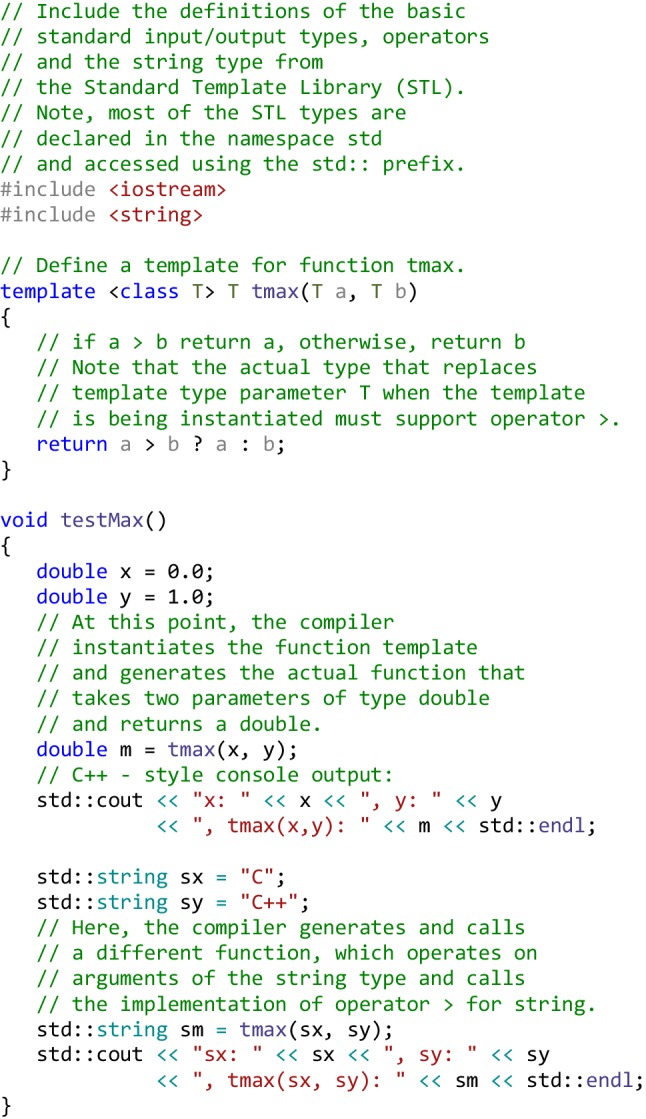


Nearly all current C++ distributions include the Standard Template Library (STL), originally developed by Alexander Stepanov, Meng Lee and David Musser [31], whose design follows the object-oriented and generic programming patterns. STL is a set of C++ template classes to provide common programming data structures and functions such as input/output streams, lists, stacks, arrays, etc., and common algorithms, such as searching, sorting, and set operations. In a sense, STL can be thought of as an essential part of C++, and nowadays C++ programmers increasingly rely on STL rather than their own “homegrown” implementations of the basic data structures and algorithms. Many C++ scientific libraries are supplied as ‘header-only’ source code libraries and heavily rely on C++ templates to make the code generalizable so it can work with many compatible data types and various options yet be as efficient as possible. For example, a widely used C++ library for linear algebra called Eigen [32] is supplied as a set of source files containing definitions of various parameterized data types and functions. C++ compilers can generate machine instructions highly optimized for speed of execution from function and class templates, but under certain code building scenarios template instantiation can introduce binary code bloat, since the compiler may create multiple instantiations of the same templated function or class that are never actually used during the program execution. Yet another notorious shortcoming of templated code is cryptic and hard-to-interpret compile-time error messages. Even a minor accidental typo somewhere in the code using templates can result in a horrific compiler error message several pages long due to very complex templated type definitions and template instantiation rules. It should also be noted that C++ template code libraries must be distributed to users as C++ source files rather than binary code libraries, which means that users of these libraries will be able to browse the source code and study its inner workings. There exist tools that can “obfuscate” C++ source code, making it intentionally hard to understand without changing its functionality, which are sometimes used by authors of closed-source software that must be delivered to customers in the form of C/C++ source files.

### An illustrative example of object-oriented and generic C++ code

To summarize this short overview of the C++ language, let us consider a somewhat more elaborate example, which illustrates concepts of object-oriented and generic programming in modern C++. The source code for this example can be obtained from Additional file 1. It can be compiled and run from the command line on most modern platforms that come with a C++ compiler supporting at least C++11. The example contains a very basic implementation of the data type (or “class”, in C++ parlance) Molecule to represent chemical structures in a program. The example is extensively commented, and the reader is encouraged to examine it closely. At the top of the class hierarchy lies the template class Graph<TNode, TEdge>, which represents an abstract graph of nodes connected by edges and implements such basic operations as adding nodes, connecting them by edges, and accessing node and edge objects. Classes Atom and Bond represent chemical atoms and bonds, respectively. Class BaseMolecule derives from Graph<Atom, Edge> and adds molecule-specific behavior to the generic graph operations. Note that, when template arguments are provided, they are substituted for the template parameters to obtain a specialization of the template, so the class Graph<Atom, Edge> is a *specialization* of the template class Graph<TNode, TEdge>. In addition to the data members and methods inherited from Graph<Atom, Edge> , BaseMolecule adds methods specific to molecules, such as the functions that add atoms and bonds with certain atom- and bond-specific properties, such as the atomic number and bond order. Class Molecule is derived from BaseMolecule and further extends it with the additional property called *name* and adds a method to compute the molecule formula. It also redefines (“overrides”) the toString method inherited from the base class. The re-defined method returns a more detailed textual description of an object of the type Molecule compared to the base class method.

A more sophisticated “real-world” C++ code example of the manipulation of chemical structures using the RDKit open-source cheminformatics library [33] can be found in the *Getting Started* section of the RDKit code repository [34]. The reader is encouraged to configure the build environment and first compile and run the simple **molecule.cpp** example from this paper, and then attempt to follow instructions in [34] to install RDKit and its dependencies, configure the build environment, build the RDKit library, and then and compile and run the example.

## Interoperability between C/C++ and other high-level languages

C and C++ are universal languages equally well suited for practically all types of coding, which still stay solidly on top of the most popular languages for system and embedded software programming, office desktop application development, and the implementation of high-performance number-crunching, image and text processing algorithms for real-time stock market data analysis, 3D animation and numerous life science applications. However, it is a very common practice to implement different parts of a program or software library in more than one programming language. There are many compelling reasons for “mixed language” development, which can be roughly split into the following two categories:

**Legacy software library reuse:** For example, there exist comprehensive high-quality software libraries for numerical analysis and linear algebra written in Fortran: BLAS [35], NAG [36], and LAPACK [37]. When developing a C or C++ application or a software module that relies on certain numerical algorithms already implemented in one or more of these mature Fortran libraries, optimized and thoroughly tested, the time and effort required to incorporate the existing Fortran modules into C or C++ code is much smaller compared to the time and effort that would be needed to translate these libraries from Fortran to C or C++ in order to develop “monolingual” code. Even though automatic converters from Fortran to C do exist, for example, *f2c* [38] and FABLE [39], the result of conversion of non-trivial Fortran code to C or C++ often leaves a lot to be desired and a substantial amount of work is usually required to clean up, debug and test the output of these automated converters.

**Coding convenience and productivity:** it is a very common scenario where the main “driver” language in which a particular program or server-side application is implemented is much better suited for a specific task than C or C++, but, in order to achieve required performance characteristics and/or implement low-level hardware access, certain critical modules have to be written in C or C++. For instance, statistical computing is ruled by R [40], MATLAB [41] is a very popular platform/language for numerical computing, a significant fraction of server-size components for various business applications are written in Java, and Python has recently climbed up to the top ranks as a general-purpose language for a wide range of applications from quick prototyping to scripting complex data processing pipelines, and to programming sophisticated large-scale server-side applications. Coding everything entirely in C or C++, even though theoretically possible, would be highly counterproductive, because C and C++ are difficult to use in read-eval-print-loop interactive environments, have a steep learning curve, and lack direct language support for certain domain-specific data types and operations (for example, C++ does not have built-in operations on matrixes and data frames found in MATLAB or R). Many cheminformatics, bioinformatics, statistical and machine learning toolkits are mostly implemented in C/C++ and provide “wrappers” to expose their programmatic interfaces to interpreted languages, such as Python, and/or virtual machine-based runtime environments, such as Java or Microsoft.NET. Typical examples are well-known and widely used CACTVS [42], RDKit [33], OpenBabel [43] and OEChem [44] cheminformatics toolkits, NumPy [45] and other packages that are part of the SciPy, a Python-based ecosystem of open-source software for mathematics, science, and engineering [46], and TensorFlow data processing and machine learning library [47], just to name a few. The computationally-intensive parts of these toolkits are mostly implemented in C and C++, with wrappers provided to make it possible to use all these toolkits in Python, and some of them in Tcl, Java and Microsoft.NET environments. The ThirdDimension Explorer (3DX) data retrieval, analysis and modeling application with “native” support for chemistry and biology developed at Johnson & Johnson Pharmaceutical Research & Development, L.L.C with a significant contribution by the author of this paper was also implemented using this approach. The front-end parts of the application were mostly written in the C# language for Microsoft.NET platform, with the core chemistry, machine learning and some high-quality graphics implemented in C++ and exposed to the.NET runtime via a wrapper interface [48].

The “reverse” or “hybrid” scenarios, where an application is largely written in C or C ++, but an interpreter for a scripting language is embedded in it to provide convenient interface for program customization and control, are also quite common. VMD (Visual Molecular Dynamics) molecular modeling and visualization computer program [49] and PyMOL molecular visualization system [50] are archetypal examples, as both include embedded Python and Tcl interpreters to allow users to run Python or Tcl scripts from within these applications to perform automated tasks and execute complicated workflows.

Mixing modules written in C/C++ and other languages (for example, Fortran or Ada) compiled into machine code for the same hardware and OS can be relatively easy, especially, if the same compiler and toolchain technology is used to build all modules and libraries comprising the target software (for example, LLVM [51] or GNU compiler collection [12]). The modern language Julia, which is quickly gaining popularity in scientific computing [52], has built-in support for calling C, C++ or Fortran code using relatively simple and straightforward syntax. However, programmatic interface between modules produced from C or C++ source code (and usually packaged as dynamically-loaded libraries on Microsoft Windows platforms or shared object libraries on Linux-based platforms) and modules in other languages which are interpreted at runtime (such as Python) or compiled into virtual machine bytecode (such as C# or Java, often called “managed” runtime environments) requires a “wrapper”. Wrapper code is usually also written in C or C++ and compiled into a shared object or dynamically linked library, which is then loaded by the host execution environment at runtime. The aim of a wrapper is to allow the calling of functions written in C or C++ and compiled into machine code from other programming languages and calling functions written in other languages from C or C++ code, passing complex data types between functions, coordination of managing memory management between C/C++ and other language runtime environments, and reusing non-trivial data types across languages. Depending on the complexity of the programmatic interface exposed by a module written in C/C++ to the host runtime environment and the type of that environment (a Python or R interpreter, Java or .NET runtime, etc.), the additional effort required to create the “wrapper” code can greatly vary from trivial (for example, exposing a small set of functions taking arguments of built-in types such as integers or floating-point number or pointers to contiguous memory blocks containing data of built-in types) to very substantial (for example, exposing an object-oriented programmatic interface with complex type hierarchies and/or depending on a large number of third-party libraries, which have to be built in a certain way in order to be compatible with the host runtime). There exist multiple tools and libraries that simplify the creation of wrapper interfaces for C/C++ code to expose it to scripting or managed runtime environments. One of the most widely used tools of this kind is SWIG [53], which is very flexible and highly configurable and can generate wrappers for a large number of host languages, such as Lua, Perl, PHP, Python, R, Ruby, Tcl, C#, Java, JavaScript, Go, Modula-3, OCaml, Octave, Scilab and Scheme. SWIG relies on manually written annotated interface definition files and requires programmers to learn the SWIG-specific interface-definition language. Another widely used C/C++ wrapper aid is the *Boost.Python* library [54], which is limited to interfacing C/C++ modules with only one—but very popular—language, Python. *Boost.Python* is part of *Boost*, which is a very comprehensive collection of free open source peer-reviewed portable C++ source libraries. As stated in the project documentation, *Boost.Python* attempts to maximize convenience and flexibility without introducing a separate wrapping language. Instead, it presents the user with a high-level C++ interface for wrapping C++ classes and functions, managing much of the complexity behind the-scenes with static metaprogramming. This library is probably the best choice for experienced C++ programmers who are also well-versed in Python. For example, a very popular open-source cheminformatics toolkit RDKit [33] is mostly written in C++ and heavily relies on *Boost.Python* in the implementation of its Python interface. An alternative to *Boost.Python* is the *pybind11* library, which offers functionality similar to that of *Boost.Python*, but is much more compact and has much fewer dependencies; however, it can only be used with modern C++ compilers that support C++11 or later standards of C++ [55].

Driven by the increasing popularity of Web-based applications offering rich functionality on par with that of their desktop counterparts but delivered seamlessly over the Web and running completely inside standard Web browsers, several methods of packaging compiled C++ code have been developed to allow its execution inside a browser, driven from JavaScript. They are not yet widely used, but the corresponding standards are emerging and look very promising. An interesting discussion with some working examples of the popular cheminformatics toolkit RDKit [33] adding interactive chemical functionality to web pages can be found in Greg Landrum’s blog [56].

The ability to package modules written in C++ in such a way that they can be accessed from common interpreted or managed runtime environments, such as Python and Java, allows a treasure trove of C/C++ code already written for all kinds of data processing needs to be reused in these environments and saves tremendous amounts of time and effort that would be required to port these libraries from C/C++ to these other languages. It also allows the implementation of performance-critical parts of software in C/C++ and compiling these parts into highly-optimized machine code for maximum performance, which is especially important for interpreted scripting languages like R and Python. However, as the famous “there’s no free lunch” adage goes, mixed-language programming adds a substantial layer of complexity to the software development process. Programs designed to run in a scripting (for instance, Python or R) or managed (for instance, Java or .NET) environment become hardware- and platform-dependent once they include modules compiled into architecture- and OS-specific machine code. For example, a program implemented in “pure” Python will run on any platform without any additional porting effort, as long as a Python interpreter for that platform is available and supports the version of Python language in which the program is written. However, if a Python program depends on a C/C++ library wrapped as a Python package, one has to find a version of that package that has been built specifically for the host hardware and operating system on which the program needs to be executed. And not only that, the package must be built separately for as many different commonly used Python implementations as practically possible. For example, a version of that package built for Python 3.6.4 MSC v.1900 64 bit (AMD64) for Microsoft Windows won’t work with Python 3.6.4 on Ubuntu Linux 18 distribution or even with the same version of Python for Windows but compiled as a 32-bit rather than 64-bit release, let alone using that module with a completely different Python implementation, for instance, IronPython for the .NET platform [57]. This tremendously complicates the package building and publishing process. One may discover that a critical package on which a particular application depends is simply not available for a specific Python implementation (for example, there is a requirement that the software must run on a Windows machine, but the dependency package is only available for Linux), or two critically important packages are incompatible between each other since they depend on different versions of some third-party shared runtime library. It also happens that the same mixed-language package behaves differently on different hosting platforms. Certain routines implemented in the package may run as expected on one platform but would crash with a core dump on some other platform, or—which is often the worst possible scenario—would produce different and non-reproducible results. This is most often caused by bugs in the C/C++ source code that are sensitive to such details of implementation as memory alignment, the size of memory pointer and certain primitive built-in data types (for example, 32-bit vs 64-bit), the availability of certain hardware features, etc. And the last but not least, there can be significant overhead with crossing the boundary and passing data structures (also known as “marshalling”) between Python or Java runtime and native machine code compiled from C/C++ when calling functions implemented in C/C++ from Python or Java and vice versa. When the performance of mixed-language code becomes an issue, it is generally advised to re-write the code to minimize the number of calls that cross the language barrier as much as possible. Using a good code profiler tool can be a great help and an eye-opening experience when working on a mixed-language code optimization. Having said that, we have to point out that the mixed-language scenarios are extremely common in scientific software development, and the advantages of the existing code reuse and substantial gain in performance that can be achieved by implementing the most critical parts of the code in C/C++ overweigh the disadvantages of the additional complexity of the mixed-language software build and distribution process.

## Conclusion: C++ as a language for scientific software development

C++ is a universal multi-paradigm imperative, object-oriented and generic programming language with great library and development tool support and a very large developer community. Modern C++ compilers produce highly optimized executable code that can very efficiently utilize hardware resources. In scientific software development, C++ is widely used to write entire software packages (including stand-alone command-line or GUI applications and server backend components), or to implement just performance-critical parts of computational algorithms of applications and packages programmed in multiple languages. An excellent review of open-source molecular modeling tools was recently published by Pirhadi et al. [58]. The companion online up-to-date catalog maintained by Koes [59] lists over two hundred toolkits and stand-alone programs for cheminformatics, molecular visualization, QSAR/ADMET modeling, quantum chemistry, ligand dynamics and free energy calculations, and virtual screening and ligand design. The catalog does not classify the software by the programming language and mentions the language only for a small fraction of programs and libraries described in it. However, since the programs listed in the catalog are open-source, the author of this paper was able to browse the respective source code repositories and collect statistics on their implementation languages. As it turned out, most packages listed in the catalog are implemented in C/C++ as the primary language (75), followed by Python (52), Java (34), Fortran (18), JavaScript (9), R (7), Pascal (1), Perl (1), Haskel (1), OCaml (1), PHP (1), Scala (1) and C# (1). Nine programs or libraries out of 52 implemented mostly in Python and three out of seven implemented mostly in R have substantial performance-critical parts written in C or C++. It is worth mentioning that Fortran still remains a popular choice in the development of software heavily relying on numerical methods, such as, for instance, programs for Ab initio calculations (11 out of the 21 listed in the catalog) and Ligand Dynamics and Free Energy calculations (7 out of 21), but many of those applications whose major parts are programmed in Fortran include some components implemented in C or C++. There is also a clear trend for newer versions of packages that were originally programmed in Fortran to be completely or partially re-written in C/C++ (quite often, with Python providing the scripting interface), or in Python (with performance-critical parts written in C/C++). Detailed analysis of the C++ usage in the areas of scientific programming not directly related to cheminformatics or computational chemistry is beyond the scope of this paper, but there has been an apparent tendency in recent years towards mixed-language programming with general-purpose scripting languages, such as Python or Julia, or domain-specific languages, such as R or MATLAB, being used to implement the majority of a stand-alone application or a software package, with performance-critical and/or hardware-dependent parts programmed in C or C++.

Even though C++ is a universal general-purpose language suitable for most types of scientific programming, it is rather difficult to learn, lacks built-in support and “shorthand” syntax for operations on common data structures such as, for example, matrices and data frames found in domain-specific languages such as R or MATLAB, and is not a good choice for interactive read-evaluate-print-loop execution mode. Typically, end-user applications or software libraries are coded in C and C++ by experienced programmers with domain expertise combined with technical skills and deep knowledge of hardware architecture. Data scientists, computational chemists, biologists and statisticians tend to use languages like Python, R or MATLAB, which are easier to learn, better suited for interactive execution, and come with complete comprehensive computing environments supporting package management infrastructure, interactive notebooks containing “live” code and graphics, and a plethora of project management and collaboration tools. However, most of these computing environments themselves are written in C and C++, and a significant fraction of reusable packages for them have critical parts programmed in C or C++. Therefore, it is fair to say that C and C++ still totally dominate scientific programming, perhaps, maybe, not in terms of the total number of lines of code written in these languages, but in terms of how many times these lines of code have been executed.

## Supplementary information

**Additional file 1.** A simple implementation of type Molecule in C++11 to illustrate the concepts of object-oriented and generic programming.

## Data Availability

Source code for all code examples included in this manuscript is either contained in the manuscript body or available as Additional files.
